# Prevalence of reported food allergies in Brazilian preschoolers living in a small Brazilian city

**DOI:** 10.1186/s13223-022-00710-1

**Published:** 2022-08-13

**Authors:** José A. da S. Correia, Adriana Azoubel Antunes, Luiz Taborda-Barata, José Laerte Boechat, Emanuel Sávio Cavalcanti Sarinho

**Affiliations:** 1 Postgraduate Program in Child and Adolescent Health (PPGSCA), Universidade Federal de Pernambuco, Av. Prof. Morais Rego, 1235 – Cidade Universitária, Recife, PE CEP: 50670-901 Brazil; 2 Department of Medicine, Faculdade Integrada Tiradentes, Jaboatão dos Guararapes PE, Brazil; 3 Faculty of Medical Sciences, Universidade de Pernambuco, Recife, PE Brazil; 4 CICS-UBI – Health Sciences Research Centre, University of Beira Interior, Covilhã, Portugal; 5 Department of Immunoallergology, Cova da Beira University Hospital Centre, Covilhã, Portugal; 6 Clinical Immunology Service, Faculty of Medicine, Universidade Federal Fluminense, Niteroi, RJ Brazil; 7 Basic and Clinical Immunology Unit, Department of Pathology, Faculty of Medicine, University of Porto and CINTESIS, Faculty of Medicine, University of Porto, Porto, Portugal

**Keywords:** Food allergies, Prevalence, Surveys, Questionnaires

## Abstract

**Introduction:**

Although the prevalence of allergic diseases, including food allergies, has increased over recent decades, relevant information on this topic is still lacking, particularly in younger children living in small cities.

**Objective:**

To investigate the prevalence of reported food allergies in preschoolers in Limoeiro/Pernambuco, Brazil.

**Methods:**

This was a cross-sectional study with preschoolers. Parents/guardians of all preschoolers enrolled at municipal schools between March and June 2019 (total of 619) were invited to complete a screening questionnaire (total of 619). Another 151 questionnaires were applied on the streets of the town. For positive responses, a second, more detailed and validated questionnaire was completed.

**Results:**

A total of 412 questionnaires were returned, of which, 47 presented a positive response to food allergies and only 29 (7.04%) identified a particular food. The most frequently reported food items were shrimp, mollusks, pork, fruit and milk. Of the 29 who identified foods, 22 responded to the detailed questionnaire, resulting in only 4 (0.97%) positive responses. Of these, two were later discarded through clinical examinations and an open oral provocation test, resulting in a final prevalence of 0.48% of confirmed food allergies.

**Conclusion:**

The prevalence of reported food allergies was lower than that described in previous studies. The most commonly mentioned foods were shrimp, mollusks and pork, with more reports of multiple food allergies, even in children who had never been previously exposed to these possible allergens, which highlights the relevance of perception in reported food allergy studies.

**Supplementary Information:**

The online version contains supplementary material available at 10.1186/s13223-022-00710-1.

## Introduction

The prevalence of food allergies has increased worldwide, over the past decades [1]. However, there is a divergence in data registered in each region. This fact may be due to, among other issues, geographical differences and regional cultural habits, the difficulty of diagnosis, and methodological inconsistencies across studies. This, therefore, makes it difficult to assess the true dimension of the food allergy problem, whether for comparative purposes, for knowledge, or for planning government actions. Several previous studies [2, 3] have shown that there is an inconsistency between family perception and expert assessment of food allergies, which may lead either to under-diagnoses or excessive diagnoses and unnecessary dietary restrictions [4]. Moreover, besides possible genetic influences, other factors such as specific eating and cultural habits of each assessed region, different perceptions of health problems by studied populations, and even the health and illness process itself may underlie divergences in the prevalence of food allergies among different urban centers, namely between larger and smaller cities.

The increased risk of serious and potentially fatal allergic reactions, in addition to the unfavorable nutritional impact and the high socioeconomic cost resulting from the use of restrictive diets are also negatively associated factors [5–7]. Children mainly develop food allergies within the first years of life, a crucial period for growth and development. Several of the most common food allergens are those that comprise the largest portion of the nutrients in children's diets. Studies comparing the growth of children with and without food allergies have reported a smaller stature among those with an allergy to cow's milk protein perceived from the second year of life [8].

There is a lack of data regarding the prevalence of food allergies and its clinical findings in children from the preschool age group, particularly in small towns [5]. It should be noted that other factors compete with food allergies, especially in populations with low socioeconomic conditions, such as food restrictions and improprieties, in addition to infectious diseases [9].

Thus, the objectives of the present study were to study the prevalence of food allergies in a small town in an underdeveloped country, due to its importance for planning, providing resources, managing and assessing health in these locations, based on the singularities [10]. Furthermore, this understanding could also shed light onto an analysis of how cultural aspects may influence the perception of families regarding food allergies. The city in which this study was carried out is located in the Northeast region of Brazil, 72 kms from the shore, has an estimated population of 56.149 inhabitants and a medium Human Development Index. In 2019, its GDP (gross domestic product) *per capita* was US$ 2.641,14 [11].

## Methods

### Study design

This was a population-based, cross-sectional study, conducted over a period of 2 years (2019–2020). Preschool children were recruited in the town of Limoeiro, in the state of Pernambuco—Brazil. A parent/guardian completed an initial screening questionnaire on adverse food reactions and food allergies (Q1), which contained simple, direct questions on sociodemographic aspects and any previous occurrence of a reaction to food after eating. When at least one food was identified as triggering reactions, the screening questionnaire was considered positive and a second, more detailed and previously validated [12] questionnaire (Q2) was completed by the researchers, in order to obtain a better characterization of these reactions. Participants were allocated by distributing questionnaires in all eleven schools within the municipal education system to those that attended preschool (a total of 619 questionnaires), and afterwards, by researchers directly approaching parents/guardians on the main streets of the town, and handing out questionnaires (a total of 151). Those who had already received a questionnaire through the schools were not included, and a total of 770 screening questionnaires were distributed. Subsequently, in order to apply the second questionnaire, participants were contacted by name in the schools or through the provided addresses.

Sampling was determined from the population with this age group residing in the municipality, which totaled 2946 children. Based on an a prevalence value of 6% reported in a previous study, for a similar age range [13] the minimum sample size estimated to obtain a 95% confidence level and a 5% margin of error, was 340 children.

### Eligibility criteria

Children were included in the study if they met the following criteria:

- belonging to the 2 to 5 years, 11 months and 29 days age group;
- living in the municipality of Limoeiro, Pernambuco.

Children were excluded from the study if their parents/guardians were under 18 years of age.

### Data analysis

The EPI INFO 7.2.2.6 programme was used to store data and statistical analyses were performed using SPSS 21.0.

Frequency distributions of the studied variables were obtained, in addition to the prevalence and the confidence intervals (95% CI) of adverse reactions to food and food allergies. Differences in proportions were analysed using Chi-Square test with Yates’s correction, and Fisher's exact test, accepting p < 0.05 as significant. Multivariate analysis of data was proposed in case there was more than one variable with p < 0.2.

## Results

### Characterisation of the population

Of the 770 screening questionnaires that were handed out (Q1), 412 (53.51%) were completed. The mean age of the children was 3.6 years (SD = 1.1), with a median of 4 years. Table 1 presents the absolute (n) and relative (%) frequencies of the following sociodemographic variables: sex, age and birth order.

**Table 1:** Sample profile of the studied population

Profile	N = 412
Sex	
Female	206 (50.0%)
Male	206 (50.0%)
Age (years)	
Two–Three ^1^	187 (45.39%)
Four–Five ^2^	225 (54.51%)
Birth Order (n = 400)	
First	196 (49.0%)
Other	204 (51%)
Profile	N = 412

Recife, 2019.

^1^ 2 years to 3 years, 11 months and 29 days

^2^ 4 years to 5 years, 11 months and 29 days

### Presence of reported food allergies

Among the 412 completed questionnaires, 47 (11.41%) had positive replies to question A (concerning adverse reactions to food or drink). Others 320 parents/guardians (77.70%) replied “No” and 45 did not know how to reply (10.9%), thereby totaling 365 (88.59%) negative replies to this question.

There were no statistically significant differences in the prevalence of reported food allergies according to sex, age or birth order, between the groups with positive and negative results in the screening questionnaire, as presented in Table 2.

**Table 2:** Cross-referencing the prevalence of reported allergy according to sex, age and birth order

Variable	Reported Allergy		P
	Yes	No/Don’t know	
Sex of child			
Female	27 (57.4%)	179 (49.0%)	0.278
Male	20 (42.6%)	186 (51.0%)	
Total	47 (100%)	365 (100%)	
Age of child			
Two–Three	24 (51, 1%)	163 (44.7%)	0.406
Four–Five	23 (48.9%)	202 (55.3%)	
Total	47 (100%)	365 (100%)	
Birth order			
First	27 (57.4%)	169 (47.9%)	0.218
Other	20 (42.6%)	184 (52.1%)	
Total	47 (100%)	353 (100%)	

Recife, 2019

### Number of allergens associated with parent/guardian-reported food allergies

Among the positive questionnaires, allergies to three or more foods were more frequently reported, as presented in Table 3. For those who reported the involvement of three or more foods, there was a more frequent association between mollusks, pork and shrimp, with 10 responses containing the three foods concomitantly (21.28% of the total positive questionnaires). However, only 29 participants were able to identify the possible foods that had caused reactions and where elegible to continue in the study. The others, who reported condiments, flavorings, colorings, which, by definition, aren't categorized as food allergies, as well as some who didn’t identify what may have caused the reaction where excluded from the following phases*.*

**Table 3:** Frequency distribution of the number of reported foods that cause allergies

Number of food items		Positive questionnaires
1	N	10
	%	21.3%
2	N	3
	%	6.4%
3 or more	N	16
	%	34.0%
Not identified	N	18
	%	38.3%
Total	N	47
	%	100%

Recife, 2019

### Food items associated with parent/guardian-reported food allergies

With regard to the food items cited by the parents/guardians as causing the adverse reaction/allergy, there was a predominance of shrimp, mollusk, pork and milk, as presented in Table 4. The fresh fruits contained in the screening questionnaire were identified in full, resulting in 7 positive replies for that food group. Among them, two (4.3%) reported an allergy to coconut, and the other types of fruits (banana, guava, avocado, apple, tomato, acai) each received one positive reply. Among the participants who identified food in full, in the “other” category, 3 (6.4%) reported a reaction to chocolate, while only one (2.1%) reported an allergy to beans.

**Table 4:** Frequency distribution of reported foods that cause allergies among the 47 positive screening questionnaires

Food		Frequency
Shrimp	N	15
	%	31.9%
Mollusk	N	15
	%	31.9%
Pork	N	15
	%	31.9%
Fruit	N	8
	%	17.0%
Milk	N	7
	%	14.9%
Peanut	N	5
	%	10.6%
Soya	N	4
	%	8.5%
Wheat	N	2
	%	4.3%
Eggs	N	2
	%	4.3%
Fish	N	1
	%	2.1%
Vegetables	N	-
	%	-
Others	N	4
	%	8.5%

Recife, 2019

### Prevalence of reported food allergies after applying the detailed questionnaire

Only 29 participants, who identified one or more foods, were invited to participate in an interview to complete the second, confirmatory, questionnaire (Q2). Of these, 22 (75.86%) agreed to participate in this stage of the study, with Q2 being applied 1 month after Q1.

Among the 22 participants, only 4 (18.1%) confirmed that the child had presented a reaction when eating certain food(s), and their response was in agreement with that presented in the first questionnaire. Of the 18 that resulted in negative replies, one confirmed a previous diagnosis by a pediatrician of cow's milk protein allergy, although at the time of the survey, the child no longer presented a reaction after ingesting milk. Considering the total number of participants who returned the completed screening questionnaire, only 0.97% continued to present a consistent response on the detailed questionnaire regarding a suspected food allergy.

### Food items and clinical manifestations described after applying the detailed questionnaire and diagnostic tests

Of the four children, with an allergic reaction after eating food, as reported by their parents/guardians, only one continued to report a reaction to multiple foods. Another child had been diagnosed cow's milk protein allergy (CMPA) by a pediatrician at the age one year, and an intact cow's milk protein-free formula had been prescribed, resulting in symptom improvement. The parent/guardian stated that she had not continued using the formula due to the high cost involved, and was currently trying to restrict cow's milk from the diet on her own, and reported a return of symptoms after any accidental exposures to this food.

**Fig. 1:**
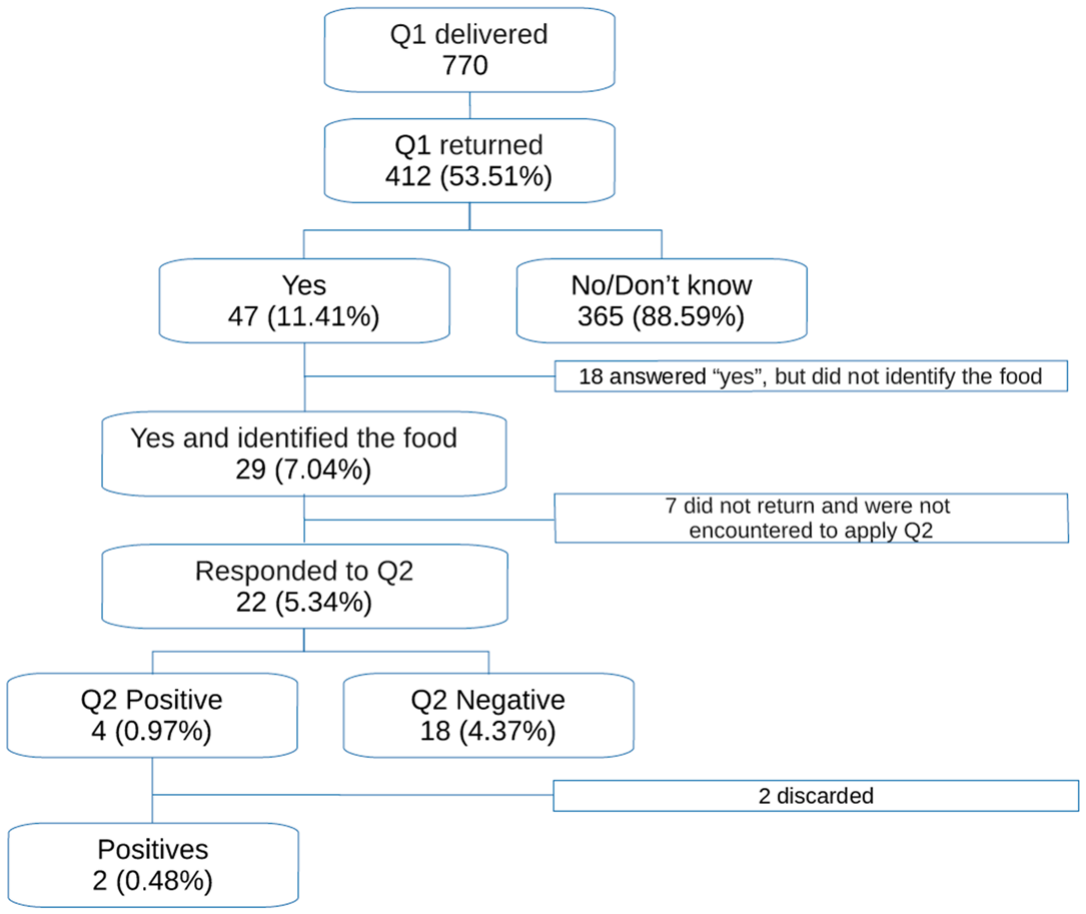
Flowchart of the research process

The three children with no previous diagnoses were invited to undergo examinations, two of whom accepted to participate. One parent/guardian reported that the child presented urticaria and digestive symptoms with a clear temporal association shortly after eating shrimp, but determination of serum shrimp-specific IgE levels was negative result (< 0.1 KU/L). Another child, with a reported reaction after ingesting acai, was skin tested but had a negative prick-prick test for this food item. These two children were advised to try these foods once again, which was performed without clinical reactions arising, and were therefore considered discarded cases. The general flowchart of collected study data is presented in Fig. 1.

## Discussion

This study is novel since it was conducted in a small town, and in a population of preschool age children, thereby having populational features which are often overlooked by many studies on food allergies. The prevalence of reported food allergies after applying the screening questionnaire was 11.7% but it dropped to 0.97% when a more thorough, confirmatory, validated questionnaire was applied.

The frequency of reported food allergies detected in our study by the screening questionnaire (11.7%) was within the range reported by other similar studies. In fact, prevalence results obtained in such studies have been quite variable ranging from 17.6%, as reported in another Brazilian study done in the southeast region with preschoolers [14], to 5.6%, as described in a Portuguese study also with preschoolers of bigger cities [15]. Both of these studies also resulted from questionnaires and involved preschool age children, while a meta-analysis with 23 selected studies described a variation in the prevalence of reported food allergies from 3 to 35% [13]. However, when we applied the second, confirmatory questionnaire (Q2), the prevalence of reported food allergy dropped to 0.97%, a value that is clearly lower than that observed in other similar studies for the same age range [14].

This clear discrepancy in the prevalence of food allergies between the screening and Q2 questionnaires suggests an overestimation of prevalence of food allergies by self-report, using short screening tests, in population studies. The huge difference observed after applying a confirmatory questionnaire is probably not due to memory bias since only a short time elapsed between applying both questionnaires. It thus seems to be based on the false perception of the presence of allergy to some foods considered unhealthy in a given culture and the low level of understanding on the part of the parents/guardians who answered the self-administered screening questionnaire. The low agreement between the findings of the screening questionnaire and the detailed questionnaire may further reiterate the importance of understanding the cultural aspects of the population in the direct approach of a patient suspected of food allergy, highlighting the importance of medical history, complementary tests and oral food challenge to confirm the diagnosis.

Various factors may account for the low prevalence of reported food allergies in our study, as detected by the Q2 questionnaire. First of all, cultural factors may play a part. With regard to cultural aspects, self-administered questionnaires enable a wide range of understandings, given the multidimensionality and complexity of the intercultural facets of communication. Among those surveyed, from a cultural viewpoint, a range of interpretations may arise in parents/guardians, after analysis of the questions asked in the questionnaire, and this may vary significantly between the population groups. In fact, issues such as knowledge regarding ancestors, religion and access to information are just some of the factors that influence the answers to these questionnaires. Thus, cultural aspects strongly influence results found by qualitative and quantitative research including self-reported data [16]. Secondly, socio-demographic factors may also have influenced our questionnaire results.

The region under study has a low human development index (0.663), with 45.2% of the population living on less than US$101 *per capita* a month. These specific characteristics of the region may have impacted on the results of this research since the variety of foods offered during childhood is also restricted [17], which may imply that children in such small cities receive less exposure to foods that are potentially more likely to trigger food allergies. Thirdly, it is also possible that the comparatively low level of reports of food allergies in the preschoolers in our study may be due to misunderstandings regarding the concepts of food allergy, since literacy levels are low in the region [11]. Finally, it is possible that the low prevalence of reported food allergy in preschoolers in our study may be due to the fact that the study region has a high prevalence (60.0% to 64.2%) of intestinal parasites for the age group studied [18, 19]. A high early exposure of a child to intestinal parasites may provide protection against allergic diseases [20].

Regarding our results on specific food allergies that were reported in the screening questionnaire, some aspects should be highlighted.

With regard to the foods mentioned in the screening questionnaire, the finding of only 1.7% prevalence of reported allergy to milk in the present study may be due to the fact that 54.51% of the children studied were aged over 4 years, an age group in which most children who present symptoms of CMPA have already developed a tolerance towards cow's milk [21].

The high prevalence of reported pork allergy (3.6%) among the study participants differs from previous literature [22]. In the region where the present study was conducted, there is a negative cultural perception about certain foods, such as pork. The main religion practiced in the region is Catholicism (80%), the second is evangelical (11%), both based on Christianity and, according to studies, with a possible influence of Judaism on new Christians [23]. There is a strong cultural bias in the local population that attributes impurity to pork. This rejection of food may therefore have led to a high number of positive responses. This fact is corroborated during the direct application of the detailed questionnaire by stating a refusal to eat pork.

Mollusks and shrimps were independently reported as causing food allergies in 15 (3.6%) participants, since they are highly perishable, and are related to frequent acute infectious complications, a fact commonly confused with allergy, which leads many families to avoid consuming these foods. Sulfite, a food additive used in the process of preparing shrimps for storage, is also usually related to adverse reactions [24], which may increase the impression of allergy to shrimp. In addition, due to its relative distance from the sea, the consumption of seafood in Limoeiro is lower than in coastal regions of the state of Pernambuco, especially among low-income families, such as those who participated in the study, due to the high costs. These factors seem to have contributed to a false impression of a food allergy to mollusks and shrimps in this study, with a subsequent denial in the detailed questionnaire off consuming these types of food.

Among fresh fruits, it is of note that allergy to these food items is more common in regions with a high incidence of pollen, due to immunologically-driven cross-reactivity, an uncommon fact in the studied area [25]. It is important to note that, among the aforementioned types of fruit, acai is rarely mentioned in food allergy studies, and is common in the cuisine of the Northern region of Brazil. This food was introduced to the Northeast region and its consumption has increased over the past 10 years. Recent research has suggested its insertion into the group of food allergens [26]. Among the aforementioned fruits, bananas and avocados are classically related to cross allergy with latex [13], while there are reports in the literature of contact dermatitis with guava [27], although with no solid references to food allergy.

Previous studies have reported that the majority of studied children react to 1 or 2 food allergens, with multiple sensitization being more uncommon [28]. In the present study, reports of allergy to multiple foods occurred more frequently among those who mentioned shrimps, pork and mollusks in the screening questionnaire, thereby emphasizing, as mentioned above, the importance of cultural perception when analysing the replies. Reports of multiple food allergies was not supported after applying the detailed questionnaires. This fact leads us, therefore, to the limitation of the screening questionnaire in separating adverse food reactions and actual food allergy, and to the importance of confirmatory tests.

Our study has various limitations. First of all, an important limitation was the fact that 24.1% of the participants who reported the presence of a reaction to food in the screening questionnaire did not continue the study. A high dropout rate is, in fact, something common in population-based studies [29], especially those involving several stages. Despite this, the sample size in our study remained sufficient to achieve statistical representativeness. Secondly, this is a report-based study of prevalence and may be influenced by memory bias [16]. Thirdly, this study was only carried out in a single city and results may not be directly generalizable to other similar cities in Brazil or elsewhere.

In conclusion, this study highlights the low frequency of reported food allergy in poor areas. Despite its limitations, it throws a light in the importance of confirming food allergy before food intake (Additional file 1) restrictions. More studies are needed to complement the data obtained.

## Main message

This article has demonstrated a low prevalence of reported food allergies in preschoolers. The particularities of the region under study, from a cultural viewpoint, and of a low social condition, in addition to hypotheses, in the light of biomedicine, explain the low frequency of food allergies for this age group in this particular region. This study highlights the importance of using validated questionnaires, for greater accuracy of the encountered data, in addition to understanding that rigorous clinical complementation with medical history, complementary exams and provocation tests are necessary to avoid excessive diagnosis, costly public measures, and nutritional and psychological repercussions. The study has demonstrated that the reality of food allergies in preschoolers and small communities may be quite different from that of large urban centers and that the specificities regarding the health situation and socioeconomic conditions of the population need to be taken into consideration.

## Supplementary Information

**Additional file 1.**Questionnaires used for screening adverse reactions to foods.

## Data Availability

All data sources of this article are available upon reasonable requesting to the corresponding author.
