# Supplementary material for: Prevalence of reported food allergies in Brazilian preschoolers living in a small Brazilian city
Source: Allergy Asthma Clin Immunol. 2022 Aug 13;18:74. doi: 10.1186/s13223-022-00710-1 (PMC9375345; doi:10.1186/s13223-022-00710-1)
Supplement: Supplementary file 1 — Additional file 1.Questionnaires used for screening adverse reactions to foods. [file 13223_2022_710_MOESM1_ESM.doc]

# QUESTIONNAIRE I

**QUESTIONNAIRE FOR SCREENING ADVERSE REACTIONS TO FOODS**

Date: ___/___/______.

Name: _______________________________________________________.

Address: _____________________________________________________________.

Date of Birth: ___/___/______. Age: ______ years.

Gender: Male Female

Brith order: firstborn

second

other

Interviewee: mother father other

Caregiver’s age: ______ years.

**A.** Does your child have a reaction or allergy to any food or drink?

Yes ( ) No ( ) Does not know ( )

**B.** Which food or drink triggers a reaction?

1. Milk …………. ( ) Yes ( ) No ( ) Does not know

2. Egg ………….... ( ) Yes ( ) No ( ) Does not know

3. Wheat …………. ( ) Yes ( ) No ( ) Does not know

4. Fish …………… ( ) Yes ( ) No ( ) Does not know

5. Soya ……….…. ( ) Yes ( ) No ( ) Does not know

6. Peanut . .....……. ( ) Yes ( ) No ( ) Does not know

7. Shrimp ………. ( ) Yes ( ) No ( ) Does not know

8. Other Shellfish .. ( ) Yes ( ) No ( ) Does not know

9. Pork .. ………… ( ) Yes ( ) No ( ) Does not know

10. Fresh fruit ……. ( ) Yes, Which?________________ No ( ) Does not know ( )

11. Vegetable …..… ( ) Yes, Which?________________ No ( ) Does not know ( )

12. Other …………. ( ) Yes, Which?________________ No ( ) Does not know ( )

# QUESTIONNAIRE II

**DETAILED QUESTIONNAIRE FOR ADVERSE REACTIONS TO FOODS**

Date: ___/___/______.

Name: _______________________________________________________.

Address: _____________________________________________________________.

Age: ______ years.

Gender: ( ) Male ( ) Female

Date of Birth: ____/____/____

Institution: ____________________________________________________________.

A. Does your child have a reaction or allergy to any food or drink?

1. ( ) Sim

2. ( ) Não

3. ( ) Does not know

B. Which food or drink triggers a reaction?

(Wait spontaneously for the answer and then read the listt)

| Food | Yes | No | Does not know |
| --- | --- | --- | --- |
| Milk |  |  |  |
| Egg |  |  |  |
| Soya |  |  |  |
| Wheat |  |  |  |
| Fish |  |  |  |
| Peanut |  |  |  |
| Shrimp |  |  |  |
| Other Shellfish |  |  |  |
| Crab |  |  |  |
| Pork |  |  |  |
| Fresh fruit |  |  |  |
| Vegetable |  |  |  |
| Other |  |  |  |

5. Fresh fruit ( ) Yes, which?_____________________________________

6. Vegetable ( ) Yes, which?___________________________________

7. Other? ( ) Yes, which?________________________________

(In case of more than one food, apply the following questions for each one)

C. When your child had the reaction, was it the first time he/she ate (or drank)

the food?

1. ( ) Yes

2. ( ) No

3. ( ) Does not know

D. How long after having eaten this food did the reaction occur?

1. ( ) Up to 2 hours / (min)

2. ( ) More than 2 hours

3. ( ) Does not know

E. Did other people eat the same food?

1. ( ) Yes

2. ( ) No

3. ( ) Does not know

F. Did those people who ate the same food have any reactions?

1. ( ) Yes, which? ________

2. ( ) No

3. ( ) Does not know

G. What type of reaction did your child have after having eaten / drunk that food/drink? (Await spontaneous response and only read the options subsequently)

| Symptoms | Yes | No | Does not know |
| --- | --- | --- | --- |
| Cough |  |  |  |
| Sneezing bout |  |  |  |
| Nasal congestion |  |  |  |
| Shortness of breath |  |  |  |
| Itchy mouth or throat |  |  |  |
| Swelling of lips, mouth or throat |  |  |  |
| Itchy eyes |  |  |  |
| Swelling of eyelids |  |  |  |
| Swelling of face, ears, hands or feet |  |  |  |
| Manchas na pele |  |  |  |
| Itchy skin |  |  |  |
| Red skin |  |  |  |
| Diarrhoea |  |  |  |
| Nausea or vomiting |  |  |  |
| Dizziness or fainting |  |  |  |
| Abdominal pain or cramps |  |  |  |
| Abdominal bloating |  |  |  |
| Blood in stools |  |  |  |
| Constipation |  |  |  |
| Other |  |  |  |

8. Other: ( ) Yes, which?_____________________________________________.

H. If this food touches the skin, your child has a reaction: redness or itching?

1. ( ) Yes

2. ( ) No

3. ( ) Does not know

I. Did your child need to go to the hospital when he/she had a reaction to this food?

1. ( ) Yes, immediately

2. ( ) Yes, one day later

3. ( ) No

4. ( ) Does not know

J. Did your child need to take any medicine at the hospital when he/she had a reaction to this food?

1. ( ) Yes, which? __________.

2. ( ) No

3. ( ) Does not know

K. Did your child need to take any medicine at home when he/she had a reaction to this food?

1. ( ) Yes, which? _________.

2. ( ) No

3. ( ) Does not know

L. After this reaction, did your child eat this food again?

1. ( ) Yes

2. ( ) No

3. ( ) Does not know

M. Did your child have the same reaction when he/she ate this food again?

1. ( ) Yes

2. ( ) No

3. ( ) Does not know

N. Did your child have other reaction when he/she ate this food again?

1. ( ) Yes

2. ( ) No

3. ( ) Does not know

O. How long have been since the fisrt reation?

P. Did your child stop eating this food after the reaction?

1. ( ) Yes

2. ( ) No

3. ( ) Does not know

Q. Has your child ever had itching or swelling or numbness in the mouth after eating some raw fruit or vegetables?

1. ( ) Yes

2. ( ) No

3. ( ) Does not know

R. Does your child have allergies?

1. Food allergy?

1. ( ) Yes 2. ( ) No 3. ( ) Does not know

2. Asthma?

1. ( ) Yes 2. ( ) No 3. ( ) Does not know

3. Rhinitis?

1. ( ) Yes 2. ( ) No 3. ( ) Does not know

4. Skin allergy?

1. ( ) Yes 2. ( ) No 3. ( ) Does not know

5. Others?

1. ( ) Yes 2. ( ) No 3. ( ) Does not know

S. Does anyone in the family have allergies?

1. Food allergy?

a) Mother

1. ( ) Yes 2. ( ) No 3. ( ) Does not know

b) Father

1. ( ) Yes 2. ( ) No 3. ( ) Does not know

c) Sibling(s)

1. ( ) Yes 2. ( ) No 3. ( ) Does not know

2. Asthma?

a) Mother

1. ( ) Yes 2. ( ) No 3. ( ) Does not know

b) Father

1. ( ) Yes 2. ( ) No 3. ( ) Does not know

c) Sibling(s)

1. ( ) Yes 2. ( ) No 3. ( ) Does not know

3. Rhinitis?

a) Mother

1. ( ) Yes 2. ( ) No 3. ( ) Does not know

b) Father

1. ( ) Yes 2. ( ) No 3. ( ) Does not know

c) Sibling(s)

1. ( ) Yes 2. ( ) No 3. ( ) Does not know

4. Skin allergy?

a) Mohter

1. ( ) Yes 2. ( ) No 3. ( ) Does not know

b) Father

1. ( ) Yes 2. ( ) No 3. ( ) Does not know

c) Sibling(s)

1. ( ) Yes 2. ( ) No 3. ( ) Does not know

5. Other?

a) Mother

1. ( ) Yes 2. ( ) No 3. ( ) Does not know

b) Father

1. ( ) Yes 2. ( ) No 3. ( ) Does not know

c) Sibling(s)

1. ( ) Yes 2. ( ) No 3. ( ) Does not know
